# Efficacy and Safety of Different Treatments for Melasma: Network Meta-Analysis of Updated Data

**DOI:** 10.3390/diseases13100316

**Published:** 2025-09-25

**Authors:** John Hang Leung, Henry W. C. Leung, Shyh-Yau Wang, Yeu-Chai Jang, Agnes L. F. Chan

**Affiliations:** 1 To Be Better One Aesthetic Clinic, Taipei City 100, Taiwan; pc1598jl@gmail.com; 2 An-Nan Hospital, China Medical University, Tainan 709, Taiwan; 070506@tool.caaumed.org.tw (H.W.C.L.); 023930@tool.caaumed.org.tw (S.-Y.W.); 3 Wan Fang Hospital, Taipei Medical University, Taipei City 116, Taiwan

**Keywords:** melasma, treatment, oral tranexamic acid, topical tranexamic acid, laser, microneedling, intradermal

## Abstract

**Background**: Melasma is a chronic, relapsing pigmented skin disease with challenging and unsatisfactory treatment outcomes. This study aims to compare the efficacy and safety of different treatments for melasma. **Methods**: We conducted a comprehensive search of PubMed and EMBASE databases to identify randomized controlled clinical trials (RCTs) for melasma treatment modalities between January 2022 and January 2025. Relative efficacy refers to the comparison of the improvement in melasma severity before and after treatment for all modalities of interest at a specific time point. The Melasma Area Severity Index (MASI) (also known as modified MASI (mMASI) or half-MASI score) was defined as the efficacy index. Safety refers to the incidence of the most common adverse events. The quality of the included trials was assessed using the GRADE method. **Results**: The analysis included 14 clinical trials with 15 treatment modalities involving 738 women who met the inclusion criteria. The mean difference in efficacy index showed that intradermal PRP (platelet-rich plasma) and intradermal PRP + oTXA (oral tranexamic acid) were the best treatment options compared with HQ4%, intradermal TXA, intradermal PRGF (plasma rich in growth factor) + HQ4 (hydroquinone 4%), followed by intradermal TXALaser (intradermal TXA + Q-switched fractional 1064-nmNd:YAG lasers). The efficacy indices of other modalities were comparable. Most treatment-related adverse events were mild, were well tolerated, or resolved with treatment. The quality of evidence was generally high. **Conclusions**: This NMA showed that intradermal PRP in combination or alone is an effective and safe treatment option for melasma. PRP may be a direction for the development of new melasma treatment options in the future, but well-designed, comprehensive, large-scale randomized controlled trials are needed to verify it.

## 1. Introduction

Melasma is a multifactorial, acquired symmetrical pigmentation disorder that usually affects the face in genetically predisposed individuals. It is more common in women and people with darker skin tones. Overall prevalence rates range widely, from 1% to 50%, because they are often calculated within specific ethnic groups and geographic areas [1,2].

The pathogenesis of this disease is not fully understood, but the cause is multifactorial, including genetic susceptibility, ultraviolet radiation, photo aging, hormonal factors, cosmetics, phototoxic drugs, skin inflammation (such as contact dermatitis and cosmetic surgery) and anti-epileptic drugs. These factors may trigger or aggravate the symptoms of melasma [3]. In addition, inflammatory mediators, oxidative stress, neuroactive molecules, and sebaceous gland cells, among others, have also been shown to be involved in the pathogenesis of melasma [4].

Sunlight exposure, particularly UV radiation, damages the DNA of epidermal cells and releases various substances, including melanocyte-stimulating hormones (MSHs) and other factors, which then bind to receptors on melanocytes, stimulating these pigment-producing cells to produce more melanin. In melasma lesions, this process is amplified by highly expressed estrogen receptors, which then stimulate melanocytes in response to sunlight exposure or other triggers (such as hormones), resulting in the darkening of the skin that is characteristic of melasma. When intraepidermal melanocytes are further activated by free radicals, the resulting unique pigmentation is the hallmark of melasma [5].

Some researchers have also suggested that ultraviolet B (UVB) radiation upregulates the expression of multiple melanocyte-specific genes and stimulates melanocytes to produce more melanin and affects abnormal interaction between melanocytes and other skin cells, further contributing to promote the production of melanin in the skin [4]. This increased melanin production leads to noticeable darkening of the skin’s epidermis and dermis. Melanogenesis is regulated by the Wnt/β-catenin, PI3K/Akt, cAMP/PKA, and SCF/c-kit-mediated signaling pathways [4].

Although there are currently no curative treatments for melasma, current knowledge about photo-protection, topical and oral therapies, and procedures such as peels, lasers, and microneedling represent the main strategies for disease control and prevention. The development of new treatment strategies for persistent hyperpigmentation focuses on modulating the pathways that drive melanin production and deposition, rather than simply reducing melanin synthesis or removing it from the skin. This approach aims to provide more lasting and effective solutions for symptoms like melasma [6].

According to a literature review, the initial treatment for melasma was topical hydroquinone (HQ), tranexamic acid (TXA) and fluocinolone acetonide 0.01% plus 4% hydroquinone and tretinoin 0.05% (modified Kligman formula; fluocinolone-based triple combination cream) (f-TCC) [2,6,7]. Due to unsatisfactory side effects of initial treatments, oral, topical and intradermal tranexamic acid (TXA) have been explored for the treatment of melasma. In the past, there has been no clear consensus on the different application forms of TXA in the treatment of melasma. Recently, there has been progress in the treatment of melasma using laser and phototherapy devices, as well as chemical peels (alone or in combination with topical medications) to improve the appearance of melasma lesions. Several studies have reported positive results with intradermal TXA [8,9]. These treatments aim to accelerate the removal of melanin and promote skin cell turnover [9].

There is now a growing consensus in the field of dermatology that combination therapy is more effective than monotherapy for treating melasma because different treatments target different aspects of melasma, resulting in a synergistic effect that makes combined treatment more effective than either treatment alone [10]. Therefore, an increasing number of randomized controlled trials (RCTs) on combination treatments for melasma are being reported, but these trials are limited to comparing two or three treatments [2]. This network meta-analysis (NMA) is the first study to attempt to compare the efficacy of 15 recently used melasma treatments (monotherapy or combination therapy) to provide dermatologists or plastic surgeons with the latest evidence on the treatment of melasma.

## 2. Methods

### 2.1. Search Strategy

We conducted a comprehensive literature search of up-to-date studies from January 2022 to June 2025 according to the Preferred Reporting Items for Systematic Reviews and Meta-Analyses (PRISMA) guidelines. The protocol has not been registered. The search limited to articles published in English, using PubMed, Embase and Cochrane Central Register of Controlled Trials databases. The detailed search strategy keywords used were “melasma” or “chloasma” and “treatment” in the title or abstract, along with filters for “randomized controlled trial (RCT)” and “controlled clinical trial”. We also manually searched for additional references to avoid potentially overlooked studies. A PRISMA flow chart illustrating this selection process is presented in Supplementary Figure S1 (online).

### 2.2. Study Selection and Data Extraction

All included studies met the following criteria: (1) randomized controlled clinical trials comparing different treatments, including monotherapy or combination therapy for melasma; (2) efficacy was measured as the mean difference (MD) in MASI, modified MASI (mMASI), or half-MASI scores before and after treatment.

Studies were excluded if they (1) contained irrelevant topics, duplicates, reviews, or comments; (2) lacked appropriate or complete data; and (3) did not report melasma-specific outcome measures. If the updated trials were published, only the latest results were included in the analysis. One author examined the final selected trials to verify their adherence to the inclusion criteria. Two independent authors (LJ and AC) assessed the data extracted from the eligible RCTs. For any controversy, a third reviewer (LH) was consulted and a consensus was reached.

### 2.3. Quality Assessment of the Included Studies

The quality of the selected RCTs was assessed using the Cochrane Collaboration’s risk of bias (RoB) version 2 assessment tool to assess the quality of each included study. Five domains are included in the revised RoB tool [11], including (1) bias due to the randomization process, (2) deviation from intended intervention, (3) missing outcome data, (4) measurement of outcomes, and (5) selection of the reported result, as well as an “overall risk of bias” judgment. Each domain was explicitly evaluated as having a low risk of bias, a high risk of bias, or some concerns [11]. Risk of publication bias was presented as a funnel plot and then assessed using Review Manager (RevMan 5) software version 5.4.1 [12].

### 2.4. Statistical Analysis and Data Synthesis

Pairwise meta-analysis was performed using RevMan 5 version 5.4.1 to estimate the overall effect size of the mean difference in MASI at the end of treatment time point. The *I^2^* test was used to assess heterogeneity in direct comparisons. If the *I^2^* value was ≤50%, a fixed effects model was selected. The efficacy was mean difference (MD) with corresponding 95% confidence intervals (CIs). Statistical significance was defined as *p* < 0.05.

Direct and indirect comparisons between treatment modalities were conducted within the Bayesian framework using STATA version 15.0 software (Stata Corp LLC., 4905 Lakeway Drive, College Station, TX, USA). The efficacy index of improved mean difference (MD) along with 95% CIs for each result was summarized. The ranking of treatments is expressed as the area under the cumulative ranking curve (SUCRA), which is a percentage that indicates the probability of the most effective treatment [13].

### 2.5. Inconsistencies and Sensitivity

Inconsistency and consistency between direct and indirect evidence were assessed by plotting the posterior mean deviations for individual data points from the inconsistency model against those from the consistency model to identify potential inconsistencies within the network.

We also performed sensitivity analyses by re-running models to compare results—excluding one study that may have been at high risk of bias—and estimating its effect.

## 3. Results

### 3.1. Characteristics of the Included RCTs

A total of 35 studies were identified through the literature search (PubMed, Embase, and Cochrane Libraries). Fourteen clinical trials with fifteen treatment modalities involved 738 women who met the inclusion criteria. Details of all of these studies are shown in Supplementary Table S1. The mean age of patients was 25 ± 6.95 to 47.2 ± 6.0 years across all studies. Six of the fourteen studies compared intradermal TXA or PRP (platelet-rich plasma) with each other and with the included treatments. Twelve of fourteen studies reported Fitzpatrick skin types ranging from II to V [14,15,16,17,18,19,20,21,22,23,24,25], while two studies did not specify skin types [26,27]. The duration of treatment evaluation was 2 to 7 months. Six studies were randomized split-face study types, while two studies were Q-switched Nd:Yag 1064 nm (QSND) laser combined with either oTXA or tTXA treatment. The remaining studies were randomized controlled trials.

**Figure 1:**
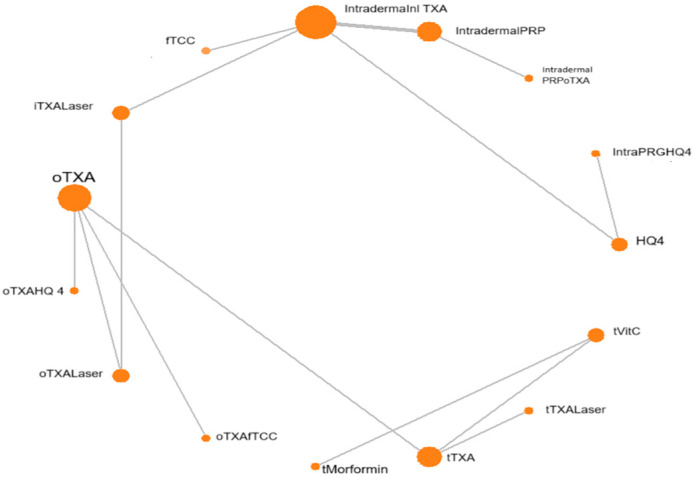
Network evidence plot for mean difference. f-TCC, fluocinolone-based triple combination cream; PRGF: plasma rich in growth factors; mTXA, microinjections (4 mg/mL); PRP, platelet-rich plasma; QSND, Q-switched 1064 laser; PIH: post-inflammatory hypopigmentation; GD: gastrointestinal discomfort.

Figure 1 presents an NMA plot, in which the size of nodes reflects the number of participants, while the line thickness indicates the number of clinical trials using the two-point treatment intervention.

### 3.2. Quality of Included Studies

**Figure 2:**
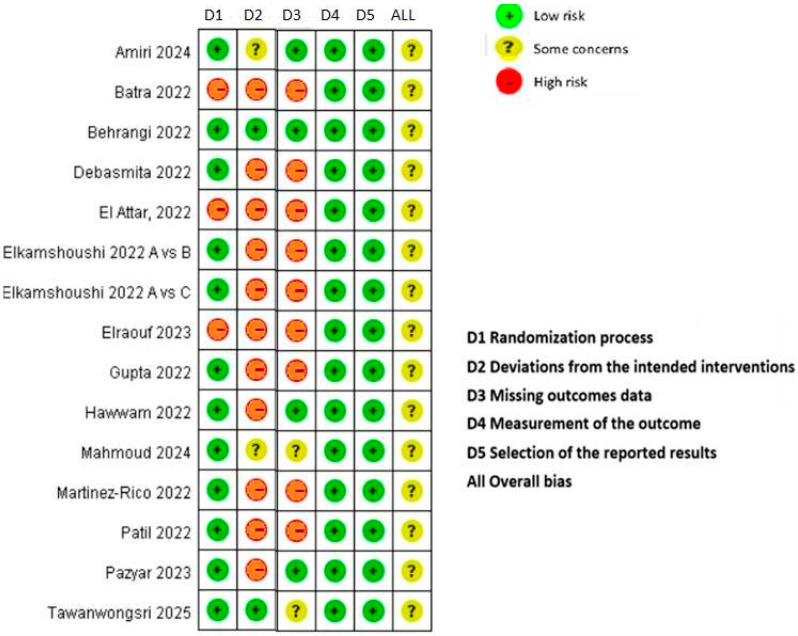
Cochrane risk of bias assessment tool version 2 for randomized trials.

Figure 1 shows the risk of bias in the 14 RCTs included in this network meta-analysis according to the criteria for risk of bias using the GRADE method. Overall, all studies had a low risk of bias in domains 4 (outcome measurement) and 5 (selection of reported outcomes), but a high risk of bias in domain 2 (deviations from the intended interventions). Only 21.4% (3 out of 14) of studies showed a high risk of bias in the randomization process. Overall, the risk of bias of the 14 included clinical trials was low (Figure 2).

The funnel plot of the risk of publication bias showed that most of the scattered points were evenly distributed on both sides of the vertical line, presenting a symmetrical distribution, except for four studies showing publication bias [14,18,20,25] (Supplementary Figure S2).

### 3.3. Network Meta-Analysis Results

#### 3.3.1. Direct Pairwise Meta-Analysis

Direct pairwise comparisons of all included studies showed that the pooled efficacy index as the mMASI score of all treatment interventions was not greater than its relative control treatment (MD [95% CI]: 0.11 [0.03, 0.18], *p* = 0.008) (Supplementary Figure S3). However, five of the fifteen treatments showed a significantly decreased mean difference in MSAI score. The most effective treatments included the following: intradermal TXA compared with topical HQ4% (MD [95% CI]: −1.30 [−2.91, 0.31]); intradermal TXA + QSND laser compared with intradermal TXA (MD [95% CI]: −1.10 [−2.07, −0.13]); intradermal TXA vs. intradermal PRP (MD [95% CI]: −0.97 [−3.67, 1.72]); oTXA + QSND laser vs. intradermal QSND laser (MD [95% CI]: −0.10 [−0.65, 0.45]); and oTXA compared with oTXALaser (MD [95% CI]: −0.06 [−3.00, 2.88]), respectively.

#### 3.3.2. Bayesian NMA

Indirect comparisons of all treatments in this NMA are presented in a league table comparing treatment efficacy in terms of the mean difference at the end of treatment. Each cell in the lower left presents the mean difference (MD) listed in the row corresponding to that column. The corresponding MD value is less than 1, which indicates that interventional treatment is beneficial (Table 1). Therefore, the results indicated that intradermal PRP showed better efficacy than intradermal TXA listed in the fifth column [MD:−1.18; 95% CI (−1.85, −0.5)] and IntraPRGFHQ4 [MD:−2.25; 95% CI (−4.06, −0.46)]. Meanwhile, efficacy of intradermal PRPoTXA is also superior to intradermal TXA [MD:−1.18; 95% CI (−1.89, −0.45)] and IntraPRGFHQ4 [MD:−2.25; 95% CI (−4.08, −0.45)]. Furthermore, oTXAfTCC was significantly superior to tTXA and tTXALaser [MD:−1.43;95% CI (−2.78, −0.08)] [MD:−1.89;95% CI (−3.44, −0.33)], while oTXAHQ4 showed a higher efficacy than tTXA [MD:−2.76;95% CI (−5.32, −0.23)] and tTXALaser [MD:−3.22;95% CI (−5.89, −0.55)]. However, vitamin C used in dermapen microneedling was inferior to all other treatment options of interest, and the efficacy of other treatment options was comparable.

#### 3.3.3. SUCRA Ranking of All Treatments Included

**Figure 3:**
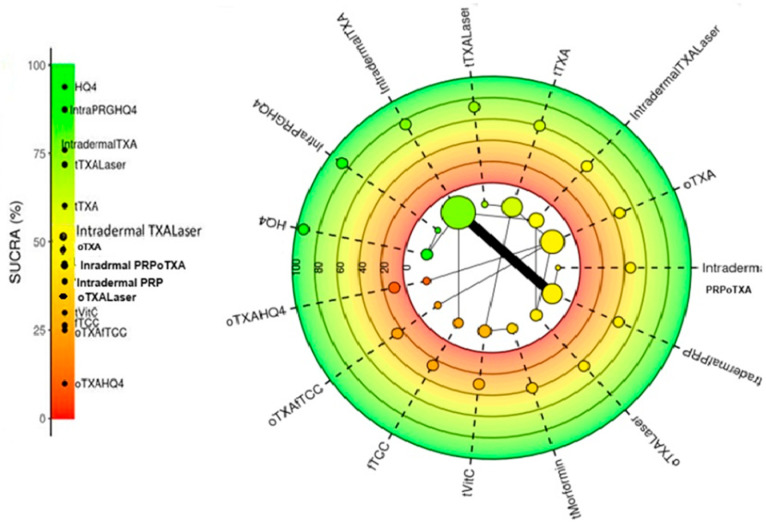
Radial SUCRA plot: Higher SUCRA values indicate higher probability of effectiveness; size of nodes represent number of participants; and thickness of lines indicate number of trials conducted. f-TCC, fluocinolone-based triple combination cream; PRGF, plasma rich in growth factors; tTXA (TXA solution transepidermal); PRP, platelet-rich plasma; QSND, Q-switched 1064 laser; MN, dermapen microneedling.

The cumulative area under the ranking curve (SUCRA) was used to rank the relative efficacy for mean difference in treatments. SUCRA values range from a high of 100% to a low of 0%. The higher the SUCRA value, the greater the probability that the treatment was better (Figure 3).

#### 3.3.4. Inconsistency and Sensitivity Analysis

An inconsistency plot for all the treatment studies is presented in Supplementary Figure S4.

This plot shows the contribution of each data point to the residual deviance from the consistent NMA model (horizontal axis) and the inconsistent model with uncorrelated mean effects (vertical axis), along with contour lines. Points on the contour lines indicate that the model fit does not improve when the inconsistent model is used, indicating that there is no evidence of inconsistency.

Sensitivity analyses showed that estimates of treatment comparisons were very similar to those of our primary analysis after excluding studies with high potential for bias. Thus, our findings are robust.

### 3.4. Adverse Effects

Most adverse events were mild and well tolerated, or resolved after the use of emollients and ointments. Pain at the injection site and transient burning sensation were the most common adverse reactions for intradermal TXA. Oligomenorrhea, a rare adverse reaction, occurred only for oTXA treatment (325 mg every 12 h). Due to limited detailed reports on adverse reactions in the included articles, the results are for reference only.

## 4. Discussion

The intractable and recurrent nature of melasma often necessitates multimodal treatment in clinical practice. This includes combining various oral or topical treatments with laser or phototherapy, intradermal as well as microneedling with or without tranexamic acid (TXA) [28].

Results of this NMA showed that the relative efficacy of topical triple combination cream fTCC was comparable to that of HQ4% alone for the treatment of MASI. The result is consistent with a previously published study, which indicated that the triple combination creams (including fluocinolone, hydroquinone and tretinoin) are generally considered more effective than HQ alone and may be safer for long-term use. However, long-term use of fTCC may cause adverse reactions such as allergic and irritant contact dermatitis, redness, burning and capillary dilation, or incomplete removal of melasma, resulting in unsatisfactory treatment results for some patients [29].

In this study, intradermal PRP and intradermal PRPoTXA were ranked third and fourth in the league table. Both modalities were superior to intradermal TXA and intradermal PRGF + HQ4 in reducing MASI scores in the treatment of melasma. Our result is supported by current evidence, which suggests that intradermal PRP can significantly reduce the severity of melasma as measured by the MASI score, particularly for long-term use. In addition, some studies have also shown that it can increase patient satisfaction [30,31,32,33,34].

For intradermal TXALaser, the mean difference in reduction in MASI score was comparable to intradermal TXA but superior to treatment with intradermal PRGFHQ4 (platelet-rich growth factor) alone. This result is supported by several studies in recently published meta-analyses [18,35,36]. The authors concluded that intradermal tranexamic acid (TXA) combined with laser therapy was more effective in reducing MSAI scores than either intradermal TXA or intradermal PRGFHQ4 alone [35]. The available evidence can explain the benefit of using lasers, which form microchannels in the skin, allowing for better penetration of TXA [35]. However, the lasers themselves may also have some effect on pigmentation. Although direct studies comparing intradermal TXA lasers with simple intradermal TXA injections are limited, in general, studies with lasers suggest they are effective for melasma [35].

Regarding intradermal PRPoTXA compared to intradermal TXA alone, recently published studies indicate that combining intradermal PRP injections with oral TXA can lead to significant reductions in melasma severity, with potentially better long-term outcomes and lower recurrence rates [34]. However, in terms of comparing intradermal PRPoTXA with intradermal PRGFHQ4 (platelet-rich growth factor + HQ4), studies have shown that both treatment options offer potential benefits as the combination of intradermal PRP and oral TXA appears to be a more effective approach for treating melasma, with reduced recurrence rates. Nevertheless, more studies are needed to directly compare their efficacy with intradermal PRGF HQ4 for the treatment of melasma, particularly on more specific cases [30,33]. The findings seem to be consistent with future treatment recommendations that a multimodal approach should be considered for the treatment of melasma, as monotherapy approaches often have limited long-term efficacy [28].

The underlying mechanisms of improvement in melasma following PRP treatment remain unknown. However, two hypothesized mechanisms have recently been proposed for the effect of PRP and PDGF on melasma, including a decrease in melanin synthesis and the augmentation of skin volume. PRP is mediated by an isoform of TGF-β (TGF-β1) and EGF. TGF-β1 induces a delay in the activation of extracellular signal-regulated kinase (ERK) and therefore indirectly inhibits melanogenesis. Additionally, EGF may inhibit prostaglandin-E2 (PGE2) expression and decrease tyrosinase enzyme activity; both are involved in the mechanism of melanin production and potentially contribute to the reduction in melasma or hyperpigmentation [37]. Platelet-derived growth factor (PDGF), released by platelets, plays a rejuvenating role in melasma treatment by increasing skin volume and stimulating the synthesis of collagen and the extracellular matrix. PDGF also indirectly contributes to the improvement in melasma by aiding angiogenesis and improving skin tone and complexion [38].

Finally, a comparison between oTXAHQ4 (oral TXA + HQ4%) and oTXALaser (oTXA + QSNDlaser) showed that both modalities were not particularly superior to HQ4% in the treatment of melasma. This supports the synergistic effect of these therapies when combined in the treatment of melasma [28].

Interestingly, the antidiabetic drug metformin has been reported to have potential effects in melasma treatment when used topically with microneedling in a recently published study [25]. Although its efficacy was not significant compared with other treatments in our study, further research is still warranted to further explore whether it can be used to treat melasma. Due to its multifaceted mechanism, including inhibiting the expression of the microphthalmia-associated transcription factor (MIF), this inhibits the transcription of several melanogenic proteins, including tyrosinase, TRP-1, TRP-2 and protein kinase C-β [39]. It also has antioxidant properties that can help reduce oxidative stress in the skin, potentially contributing to the improvement in melasma. Another hypothesized metformin mechanism is its insulin-sensitizing properties, as insulin resistance may play a role in some cases of melasma. Its specific effects on melasma may be related to the insulin signaling pathway [40].

Safety data of treatment provided from studies included in this NMA are limited. The most common adverse events of treatment with oTA are usually mild, and include gastrointestinal discomfort, musculoskeletal pain and oligomenorrhea [14,19,26]. In a recently published study, the authors reported that oTA is safe for the treatment of melasma at a dose of 325 mg bid over a 4-month treatment period, and that combined use of oTA and f-TCC enhances treatment efficacy in patients with severe melasma [19].

The most common adverse events of intradermal PRP and TXA are injection pain, transient mild pruritus and erythema [22,23]. Therefore, both TXA and PRP intradermal are considered effective and safe treatments for melasma. The risk of serious adverse reactions is low, but it is important to screen patients for their risk nonetheless.

The advantage of this NMA is its low risk of bias because most studies were randomized controlled trials. However, limitations remain. First, there is insufficient comparative evidence due to the lack of detailed safety data for statistical analysis. Second, the sample size of the included studies was small, and most of the studies were not double-blind, so this NMA may have selection and implementation biases. However, robust sensitivity analysis results can minimize limitations.

## 5. Conclusions

In conclusion, the optimal new treatment strategy for melasma may be a multimodal approach, including effective photo-protection and a combination of different treatment modalities targeting melanin synthesis, the anti-inflammatory milieu, aging, and vascularity. More new treatment modalities, such as oral or topical TXA combined with intradermal TXA or PRP, are expected to gain additional evidence in the near future from well-designed, methodologically sound, large-sample randomized controlled trials. Additionally, future research should explore more personalized and effective methods to improve patient satisfaction with care and treatment outcomes.

## Figures and Tables

**Table 1:** League table for all treatments included.

**fTCC**														
−3.49	**HQ4**													
(−6.18, 0.79)														
−1.01	2.47	**Intradermal PRP**												
(−3.23, 1.23)	(0.72, 4.23)													
−1.01	2.46	0	**Intradermal PRPoTXA**											
(−3.25, 1.25)	(0.7, 4.24)	(−0.25, 0.25)												
−2.19	1.29	−1.18	−1.18	**Intradermal TXA**										
(−4.32, −0.05)	(−0.33, 2.92)	(−1.85, −0.5)	(−1.89, −0.45)											
−1.08	2.4	−0.07	−0.07	1.1	**Intradermal TXALaser**									
(−3.43, 1.26)	(0.5, 4.28)	(−1.25, 1.11)	(−1.27, 1.14)	(0.14, 2.07)										
−3.28	0.21	−2.25	−2.25	−1.08	−2.19	**Intra PRGFHQ4**								
(−5.99, −0.55)	(−0.18, 0.6)	(−4.06, −0.46)	(−4.08, −0.45)	(−2.76, 0.58)	(−4.11, −0.25)									
−0.92	2.57	0.1	0.09	1.3	0.17	2.37	**oTXA**							
(−4.83, 2.97)	(−1.07, 6.22)	(−3.23, 3.43)	(−3.24, 3.44)	(−1.95, 4.56)	(−2.92, 3.27)	(−1.29, 6.02)								
−0.03	3.46	0.99	0.99	2.19	1.05	3.25	0.89	**oTXAfTCC**						
(−4.02, 3.94)	(−0.28, 7.19)	(−2.43, 4.43)	(−2.44, 4.43)	(−1.14, 5.56)	(−2.14, 4.28)	(−0.51, 7.01)	(0.05, 1.73)							
1.31	4.79	2.32	2.32	3.53	2.4	4.58	2.22	1.33	**oTXAHQ4**					
(−3.2, 5.82)	(0.48, 9.09)	(−1.71, 6.36)	(−1.71, 6.38)	(−0.44, 7.52)	(−1.46, 6.25)	(0.25, 8.9)	(−0.09, 4.54)	(−1.13, 3.81)						
−0.98	2.5	0.03	0.03	1.23	0.1	2.28	−0.07	−0.96	−2.3	**oTXALaser**				
(−3.39, 1.43)	(0.54, 4.46)	(−1.27, 1.33)	(−1.29, 1.35)	(0.13, 2.34)	(−0.45, 0.65)	(0.28, 4.28)	(−3.13, 2.98)	(−4.14, 2.2)	(−6.11, 1.52)					
−0.37	3.11	0.64	0.63	1.85	0.71	2.9	0.54	−0.35	−1.68	0.61	**tMorformin**			
(−4.93, 4.18)	(−1.24, 7.45)	(−3.45, 4.72)	(−3.45, 4.73)	(−2.17, 5.88)	(−3.19, 4.63)	(−1.47, 7.26)	(−1.83, 2.9)	(−2.86, 2.14)	(−4.99, 1.6)	(−3.26, 4.47)				
−1.46	2.03	−0.45	−0.45	0.76	−0.37	1.82	−0.54	−1.43	−2.76	−0.47	−1.08	**tTXA**		
(−5.49, 2.59)	(−1.76, 5.82)	(−3.92, 3.06)	(−3.94, 3.06)	(−2.66, 4.19)	(−3.65, 2.93)	(−1.99, 5.63)	(−1.59, 0.51)	(−2.78, −0.08)	(−5.32, −0.23)	(−3.7, 2.78)	(−3.21, 1.05)			
−1.9	1.57	−0.89	−0.89	0.31	−0.82	1.36	−0.99	−1.89	−3.22	−0.92	−1.54	−0.45	**tTXALaser**	
(−6.02, 2.2)	(−2.31, 5.45)	(−4.46, 2.69)	(−4.47, 2.71)	(−3.2, 3.84)	(−4.18, 2.56)	(−2.53, 5.25)	(−2.32, 0.32)	(−3.44, −0.33)	(−5.89, −0.55)	(−4.24, 2.42)	(−3.78, 0.74)	(−1.24, 0.34)		
−0.26	3.22	0.75	0.74	1.96	0.82	3.01	0.65	−0.24	−1.57	0.72	0.11	1.19	1.65	**MNVitC**
(−4.81, 4.29)	(−1.13, 7.56)	(−3.34, 4.83)	(−3.34, 4.85)	(−2.05, 5.99)	(−3.08, 4.74)	(−1.35, 7.37)	(−1.72, 3.02)	(−2.74, 2.25)	(−4.88, 1.72)	(−3.15, 4.58)	(0.02, 0.2)	(−0.94, 3.31)	(−0.63, 3.9)	

Annotation: Data are presented as mean differences and 95% confidence intervals (CI) at the end of treatment. Mean differences < 1 favor treatments. Each cell in the lower left gives the mean difference in the column relative to the row. Abbreviations: f-TCC, (fluocinolone-based triple combination cream: fluocinolone acetonide 0.01% +hydroquinone 4%+ tretinoin 0.05%); PRGF: Plasma Rich in Growth Factors; mTXA (microinjections TXA); PRP, Platelet-rich plasma; QSND = (Q-switched 1064 Laser); MN = (dermapen micro needling); tTXA (TXA solution transepidermal).

## Data Availability

The authors declare that all data and materials used and analyzed in this study are contained within the paper and supplementary files (Supplementary Materials).

## Supplementary Materials

The following supporting information can be downloaded at: https://www.mdpi.com/article/10.3390/diseases13100316/s1, Figure S1: The flow chart summarizing the process for the identification of the eligible randomized controlled trials; Figure S2: The funnel plot from this study demonstrated that the majority of scatter points were evenly distributed on both sides of the vertical line. 4 studies fell outside the plot, demonstrating publication bias. Figure S3: Forest plot of pairwise direct comparison of treatments for improvement of ΔMASI at the end of therapy. The pooling results are beneficial to the comparator. Figure S4. Inconsistency plots for efficacy. Plot of individual data points for the consistency model (horizontal axis), and the inconsistency model (vertical axis), along with the line of equality. Table S1: Summary of estimated parameters and AIC/BIC values of PFS and OS models.
