# Supplementary material for: Efficacy and Safety of Different Treatments for Melasma: Network Meta-Analysis of Updated Data
Source: Diseases. 2025 Sep 25;13(10):316. doi: 10.3390/diseases13100316 (PMC12562867; doi:10.3390/diseases13100316)
Supplement: Supplementary file 1 [file diseases-13-00316-s001.zip › Supplementary Figure S1- S4.pptx]

## Slide 1
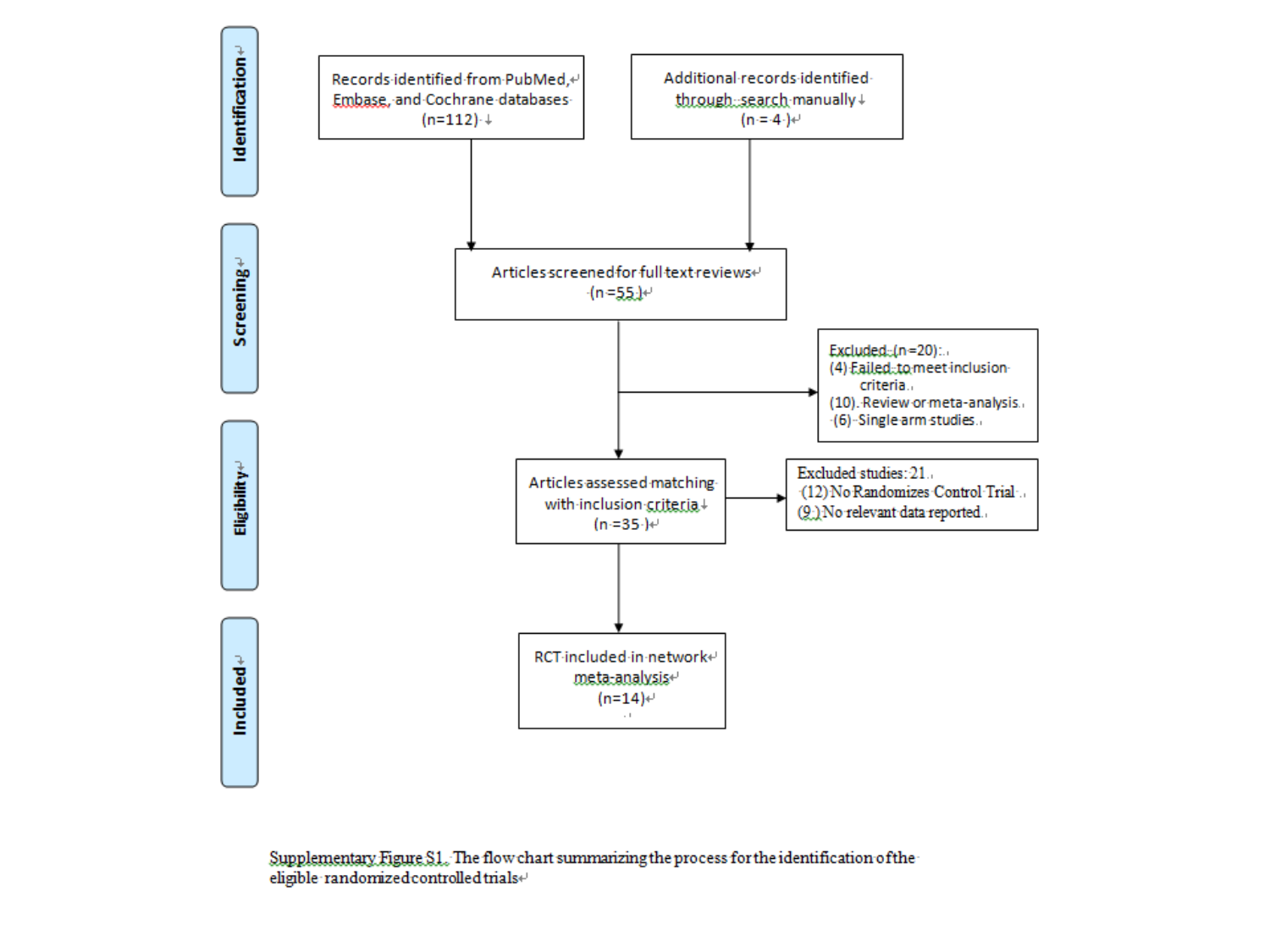

## Slide 2
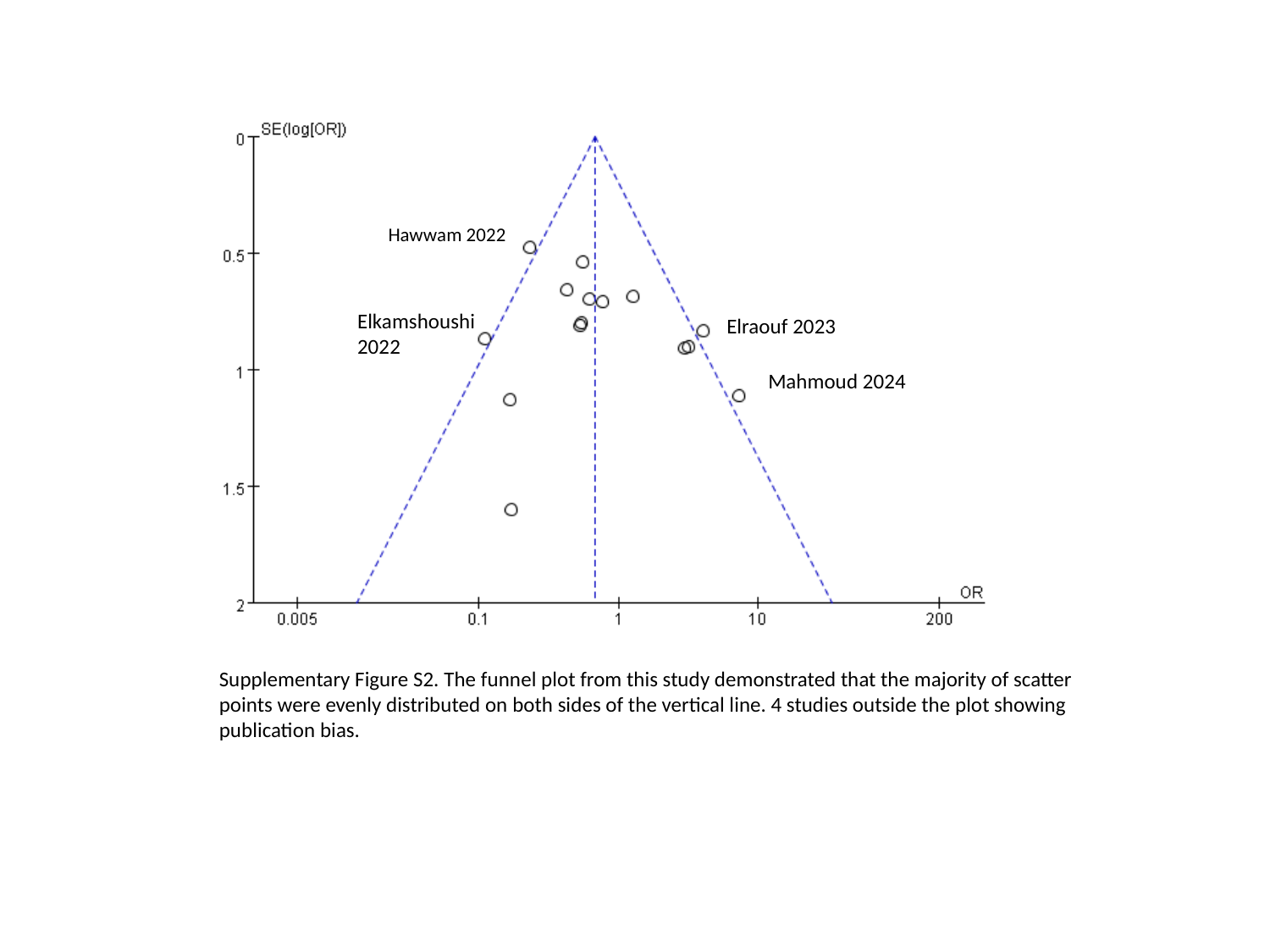

Hawwam 2022
Elkamshoushi
2022
Elraouf 2023
Mahmoud 2024
Supplementary Figure S2. The funnel plot from this study demonstrated that the majority of scatter
points were evenly distributed on both sides of the vertical line. 4 studies outside the plot showing publication bias.

## Slide 3
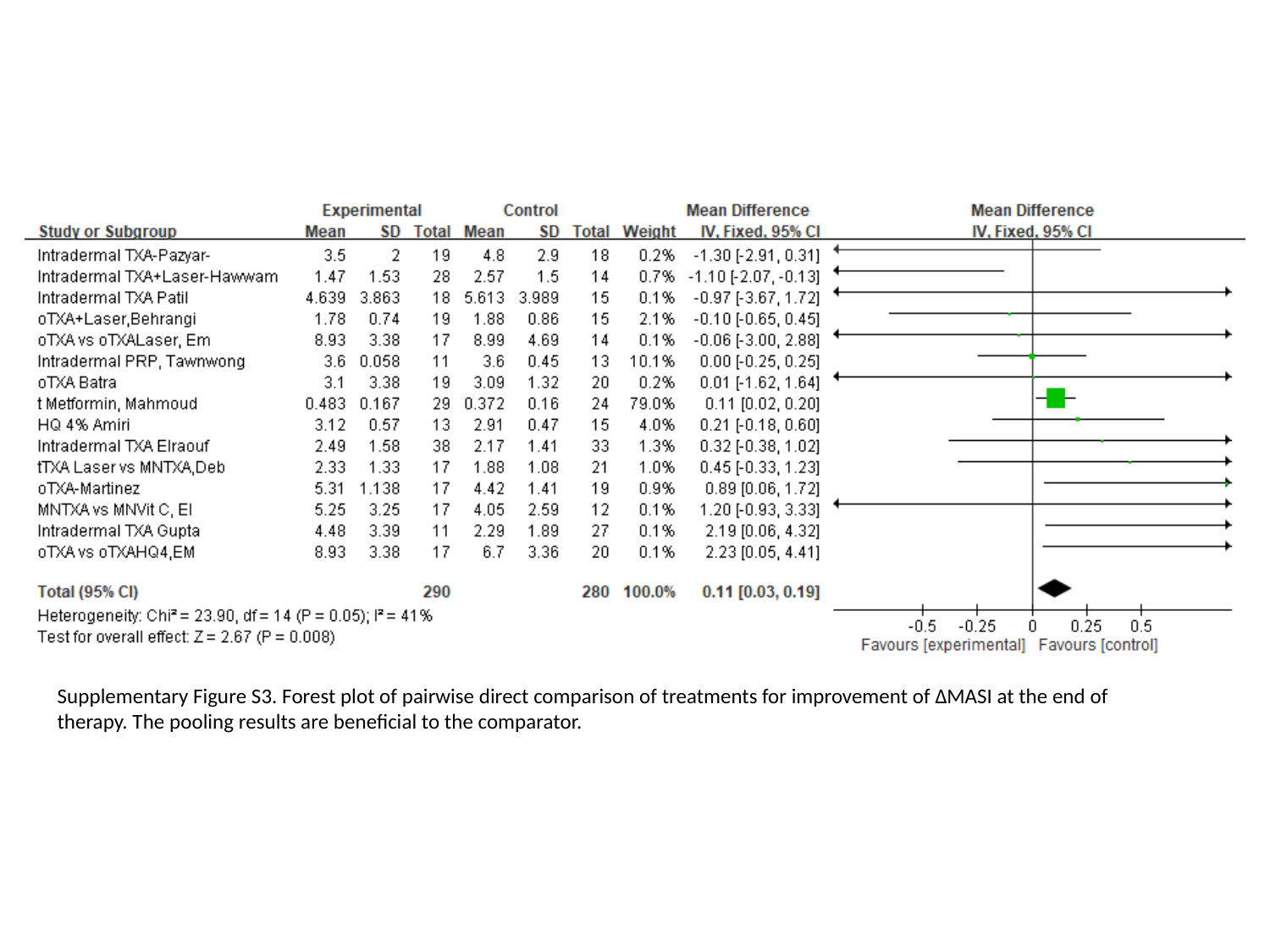

Supplementary Figure S3. Forest plot of pairwise direct comparison of treatments for improvement of ΔMASI at the end of therapy. The pooling results are beneficial to the comparator.

## Slide 4
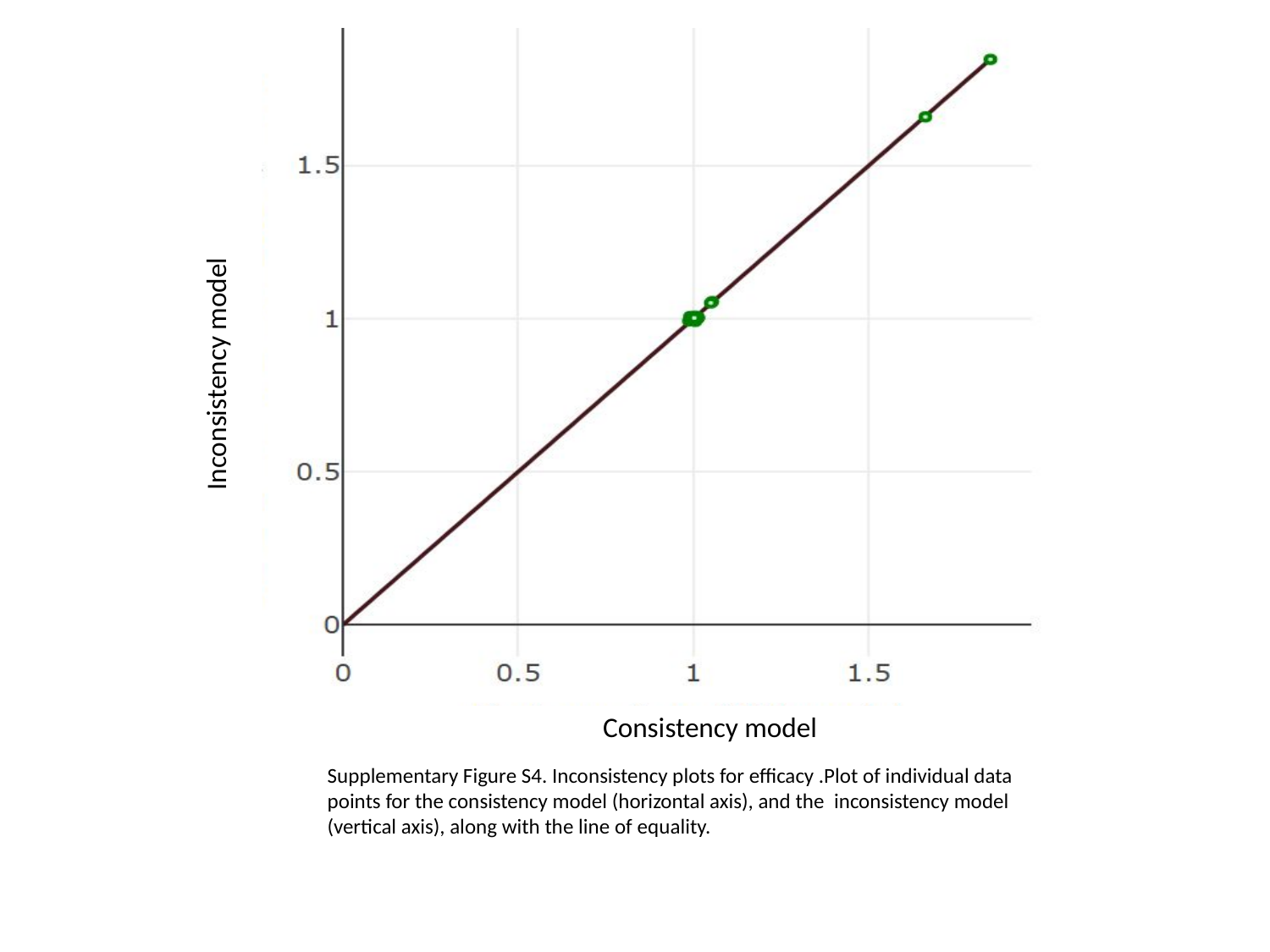

Inconsistency model
Inconsistency model
Consistency model
Consistency model
Supplementary Figure S4. Inconsistency plots for efficacy .Plot of individual data points for the consistency model (horizontal axis), and the inconsistency model (vertical axis), along with the line of equality.
