# Supplementary material for: Efficacy and Safety of Different Treatments for Melasma: Network Meta-Analysis of Updated Data
Source: Diseases. 2025 Sep 25;13(10):316. doi: 10.3390/diseases13100316 (PMC12562867; doi:10.3390/diseases13100316)
Supplement: Supplementary file 1 [file diseases-13-00316-s001.zip › Supplementary TabS1. Characteristics of included studies 0928.pdf]

Supplementary Table 1. Characteristics of included studies

Studies	Study design	Patient N (A/B/C)	Age (mean/yr) (A/B/C gr.)	Skin Phenotype	Duration Tx evaluation (month)	Mean MASI Score reduction at duration evaluation (months)	Outcomes Pt no.(%)	Interventions		Adverse effects (AE) Patient no (A/B/C)group.
								Case group	Control group	
1,Elkamshoushi 2022(Egypt)[14]	RCT	20/20/20	35.10 ± 8.44<br>32.40 ± 8.62<br>31.85 ± 8.31	III, IV	6	A:8.93 ± 3.83<br>B:6.70 ± 3.36<br>C:8.99 ± 4.69	10 (50)<br>18(90)<br>14(70)	A: oTXA 250 mg twice daily	B: oTXA+tHQ 4% once at night<br>C: oTXA+Laser (Q-switched 1064)(QSND)	A. Gastritis (3)<br>B:Itching and irritation(13), Eythema (9)<br>C: Itching and irritation(4); PIH (3)
2.Batra 2022 (India)[15]	RCT	20/20	25 ± 6.95	NA	6	2.56 ±1.95/ 3.09 ± 1.32	14 (70)<br>15 (75)	oTXA 250 mg twice daily	Transepidermal TXA (4 mg/mL)	A: Epigastric discomfort (2)<br>B: Injection site pain (7)
3.Behrangi 2022 (Iran)[16]	Double- blinded RCT	21/20	41.88 ± 5.53	I, IV	6	1.69 ±0.7/1.82 ± 0.86	19 (90)<br>15 (75)	oTXA 250 mg+Laser (QSND) every 2 weeks per visit.	tTXA (microinjection) +Laser (QSND)	A:GI (4); Headache (0);HM (1)<br>B:GI (1);Headache(1)HM (1)<br>Injection site pain(4)
4.Debasmita 2022 (India)[17]	RCT	30/30	A: 38.2 ± 7.65<br>B: 37.6 ± 7.77	NA	7	5.12 ± 2.66/2.33 ± 1.33	17 (57)<br>21(70)	Laser (QSND)+tTXA 3%<br>5 monthly sessions	tTXA 3% microneedling<br>5 monthly sessions of	A:Burning sensation (6), Pain (4)<br>Erythema (2)<br>B:Burning sensation (4), Pain (8)<br>Erythema (6)
5.El Attar, 2022 (Egypt)[18]	Split-face randomized uncontrolled	A/B:20/20	39.45 ± 6.95	III. IV	3	5.25± 3.25/4.05±2.59	15 (55%)<br>17(45%)	tTXA, microneedling every 2 weeks for 6 sessions	Vitamin C, microneedling Every 2 weeks for 6 sessions	Minimlal side effects
6.Gupta 2022 (India)[19]	RCT open label	A/B:29/30	A:36.69 ± 10.12<br>B:35.3 ± 7.01	III, IV, V	3	4.48±3.39/2.29±1.89	24 (83)<br>29(96)	Intradermal tranexamic acid 4 mg/mL of every weeks for 10 weeks	fTCC (Topical Kligman's therapy) daily for 10 weeks or till complete clearance.	A:Injection pain, transient burning (29)<br>B: Erythema (9),Burning (6)=15<br>PIH (5)
7.Hawwam 2022 (Egypt)[20]	Split randomized single blinded	A/B:40/40	37.35 ± 4.66	III, IV, V	6	A:3.04 ± 1.87 (left side)<br>B:1.22 ± 1.09 (Right side)	14 (35)<br>28 (70)	Intradermal tranexamic acid	Intradermal TXA+Laser (QSND)	Mild erythema after laser treatment and mild pain from injection (No data)
8.Raza 2022 (Pakistan)[22]	non-randomized Split face study	A/B: 30/30	20–55	IV	2	A: 4.81±3.25<br>B: 5.12±3.08	26 (87)<br>25 (82)	TXA, Micro-needling (500 mg/5 mL ampoule), 3 biweekly sessions	20% Vit C, Micro-needling 3 biweekly sessions	NA
9.Nasimi, 2022 (Iran)[23]	Split randomized single blinded	A/B: 29/29	36.85 ± 6.18	II–IV	6	A:2.46 ± 1.56<br>B: 3.39 ± 2.31	21 (72)<br>20 (69)	Erbium-YAG Laser+ Kligman's formula	Kligman's formula alone	Transient and mild erythema and scaling
10.Martinez-Rico 2022 (Mexico)[24]	RCT	A/B:22/22	40.45±3.88/ 42.82 4.90	II, III, IV, V,	4	A: 8.61±2.61 (4 months)<br>B:6.65±5.87	17(76.9)<br>19(85.1))	oTXA(325 mg every 12 h) + fTCC	oTXA (325 mg every 12 h)	A/B: Oligomenorrhe (0/7), GD(0/2);Burning (0/11) Erythema (0/8) Xerosis (0/3)
11.Elraouf 2023 (Egypt)[25]	Split-face prospective study	A/B:40/40	39.20 ± 5.22	III -IV	3	A: 2.49 ± 1.58<br>B: 2.17 1.41	38 (95)<br>33 (83)	Intradermal TXA(4 mg/ml)	Intradermal PRP	TXA: Injection pain (25), Erythema(22)<br>PRP: Injection pain (3), Erythema(13)
12.Patil 2022 (India).[26]	Prospective, randomized, open label	A/B: 20/20	A: 33.94 ± 8.34<br>B: 34.47 ± 5.998	IV, V	6	A: 10.77 ± 8.87<br>B: 12.66 ± 7.52	18 (90)<br>15 (75)	Intradermal TXA(4 mg/ml)	Intradermal PRP	Mild pruritus and erythema (no data)
13.Pazyar 2023 (Iran)[28]	Double blinded RCT	A/B:24/24	A:33.7 ± 6.1<br>B:35.9 ± 5.2	II-.V	6	A: 3.5±2.0<br>B: 4.8±2.9	A:15 (63)<br>B: 11 (46)	A: Intradermal TXA 100mg/ml	B: Hydroquinone 4% topically every night	mild degrees of burning pain at the injection site.

Remarks: f-TCC, (fluocinolone-based triple combination cream: fluocinolone acetonide 0.01% +hydroquinone 4%+ tretinoin 0.05%); Kligman's formula (4.0% hydroquinone+ 0.1% dexamethasone+3% vitamin C in the base of cold cream); tTXA (Topical or Localized microinjections (4 mg/mL); PRP, Platelet-rich plasma; QSND=(Q-switched 1064 Laser)

PIH: Post-inflammatory hypopigmentation; GD: Gastrointestinal discomfort
